# A Successful Failure: Missing the MDG4 Target for Under-Five Mortality in South Africa

**DOI:** 10.1371/journal.pmed.1001926

**Published:** 2015-12-22

**Authors:** Peter Byass, Chodziwadziwa W. Kabudula, Paul Mee, Sizzy Ngobeni, Bernard Silaule, F. Xavier Gómez-Olivé, Mark A. Collinson, Aviva Tugendhaft, Ryan G. Wagner, Rhian Twine, Karen Hofman, Stephen M. Tollman, Kathleen Kahn

**Affiliations:** 1 MRC-Wits Rural Public Health and Health Transitions Research Unit (Agincourt), School of Public Health, Faculty of Health Sciences, University of the Witwatersrand, Johannesburg, South Africa; 2 Umeå Centre for Global Health Research, Division of Epidemiology and Global Health, Department of Public Health and Clinical Medicine, Umeå University, Umeå, Sweden; 3 INDEPTH Network, Accra, Ghana; 4 Department of Global Health and Development, Faculty of Public Health and Policy, London School of Hygiene and Tropical Medicine, London, United Kingdom; 5 PRICELESS/PEECHi, School of Public Health, Faculty of Health Sciences, University of the Witwatersrand, Johannesburg, South Africa

## Abstract

Reflecting on under-five mortality, Peter Byass and colleagues consider how some countries may fail to meet millennium development goal targets despite making considerable advances.

**Summary Points**

- Millennium Development Goal (MDG) “success” is based on relatively arbitrary targets, yet countries are coming under close scrutiny as the 2015 deadline approaches and the world’s focus moves on to new Sustainable Development Goals.
- Some countries may fail to meet MDG targets, for example, because of low baseline figures, despite making considerable advances in mortality reduction.
- In South Africa, under-five mortality was already low in 1990 as democracy advanced and the HIV epidemic emerged; thus, despite considerable mortality reduction in recent years, there was no appreciable net change from start to end of the MDG period.
- Understanding changes in childhood mortality depends on having detailed data over time, which can be interpreted in a more nuanced way than simply the achievement of arbitrary targets.

## What Is “Success” in Terms of Reducing Under-Five Mortality?

When generic criteria to assess achievements are formulated long in advance of endpoints, unforeseen consequences may follow in particular instances. The Millennium Development Goals (MDGs) were fairly arbitrarily defined to be measured over the period from 1990 to the end of 2015, and now countries are being widely classified as meeting or failing those targets [1]. Achieving the MDG4 goal of reducing mortality in children under five years of age by two-thirds was always likely to be very difficult for some countries, particularly those with low mortality in 1990 [2]. The relevant, newly adopted third Sustainable Development Goal (SDG) now calls for under-five mortality to fall below 25 per 1,000 live births by 2030 [3], maintaining a global focus on achieving low childhood mortality.

**Fig 1:**
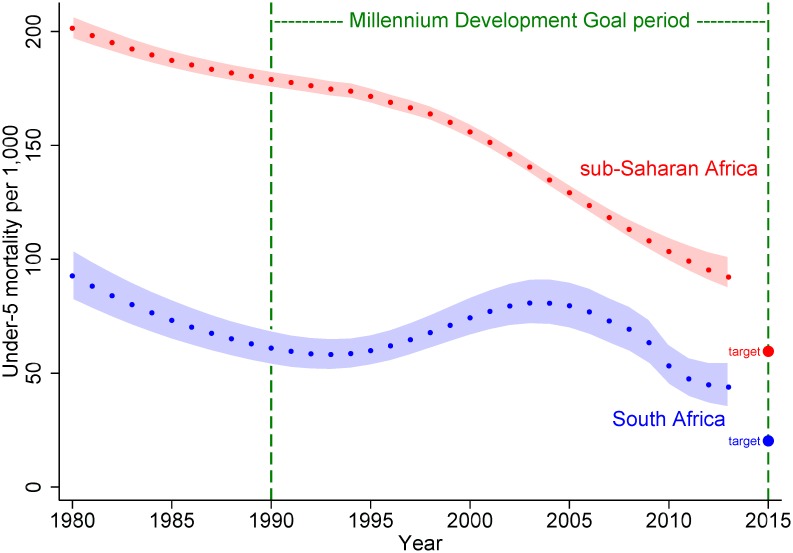
Under-five mortality estimates (with 90% uncertainty bounds) from UNICEF [4] for sub-Saharan Africa and South Africa from 1980 to 2013, together with respective MDG4 target levels for 2015 (two-thirds reduction from 1990).

Taking South Africa as an example, under-five mortality for the country in 1990 was the lowest in sub-Saharan Africa. But the intervening period has been pivotal, as the transition to democracy coincided with the start of the HIV pandemic. South Africa’s under-five mortality trajectory from 1980 to 1990 was effectively on track for the 2015 target, until a sharp rise in mortality was observed in the mid-1990s, as shown in Fig 1, using mortality estimates from UNICEF [4]. Nevertheless, South African child mortality levels remained substantially lower than in sub-Saharan Africa as a whole, despite neither the country nor the region being on track for their respective 2015 targets, and very rapid reductions in mortality were estimated for South Africa around 2010. Thus, South Africa apparently made progress before 1990, despite the iniquitous policies of the apartheid regime, and went on to record considerable reductions in child mortality during the latter part of the MDG period.

## Understanding the Detail behind Reported Mortality Changes

National mortality estimates such as those in Fig 1 cannot provide explanatory insights. Understanding MDG outcomes ideally means accessing detailed, consistent, and reliable data from the 1990 baseline onwards. For South Africa, individual-level data over the MDG period are scarce, despite the national requirement, in principle, for all deaths to be registered [5]. Particularly for causes of death, registration has sometimes been patchy and unreliable. At certain phases of the HIV/AIDS epidemic, substantial stigma attached to AIDS as a cause of death brought an unwillingness to see it on death certificates. National statistics and estimates therefore have to be regarded as being of mixed detailed validity [6].

A complementary source is detailed individual data from specific surveillance sites. Although these data cannot be demonstrated to represent national levels, they permit consideration of mortality in a defined entire population, irrespective of health services utilisation or place of death, and, where such surveillance has proceeded consistently over a long period, provide an opportunity to evaluate trends in real time [5]. One such site in South Africa is the Agincourt Health and socio-Demographic Surveillance System (HDSS) (www.agincourt.co.za) [7], which has collected data consistently over the period 1992 to 2013, spanning some of the critical transitions in the country. The site is located in rural northeastern South Africa, close to the Mozambican border.

The site data cover a subdistrict population living in areas previously designated as black Bantustans or “homelands,” with many families including temporary migrant workers who spend much of their time away from home, working particularly in the mining and service sectors. Households typically subsist on a combination of migrant remittances, small-scale agriculture, local (often governmental) employment, social transfers and trading. The population surveillance in Agincourt was based on annual visits in which all households were asked about residents’ details, and any deaths since the previous round were followed up with verbal autopsies (structured interviews with relatives or other witnesses of deaths to ascertain probable causes). Initially, a locally devised verbal autopsy instrument was used, later aligned with the WHO 2012 verbal autopsy standard [8], and the earlier data were post-processed into the same format, allowing cause of death to be determined automatically and consistently over the whole period using the InterVA-4 probabilistic model [9]. The Agincourt study site was one of the founder members of the INDEPTH Network (www.indepth-network.org) [10], an umbrella organisation for many such sites across Africa and Asia. Overall mortality trends in Agincourt for 1992 to 2011 have been presented [11] as part of an INDEPTH multi-site collection of work on cause-specific mortality [12].

## What Can Be Learnt from Detailed Individual Data?

A total of 1,997 deaths among children under five years of age were documented in the Agincourt site from 1992 to 2013, over 202,666 person-years of observation. Thus the overall mortality rate was 9.9 per 1,000 person-years, corresponding to under-five mortality of 62 per 1,000 live births. During the first year of life there were 1,149 deaths over 40,063 person-years, a rate of 28.7 per 1,000 person-years, corresponding to infant mortality of 36 per 1,000 live births. Of the deaths in infancy, the neonatal period accounted for 301 deaths over 3,049 person-years, a rate of 98.7 per 1,000 person-years, corresponding to neonatal mortality of 9 per 1,000 live births.

**Fig 2:**
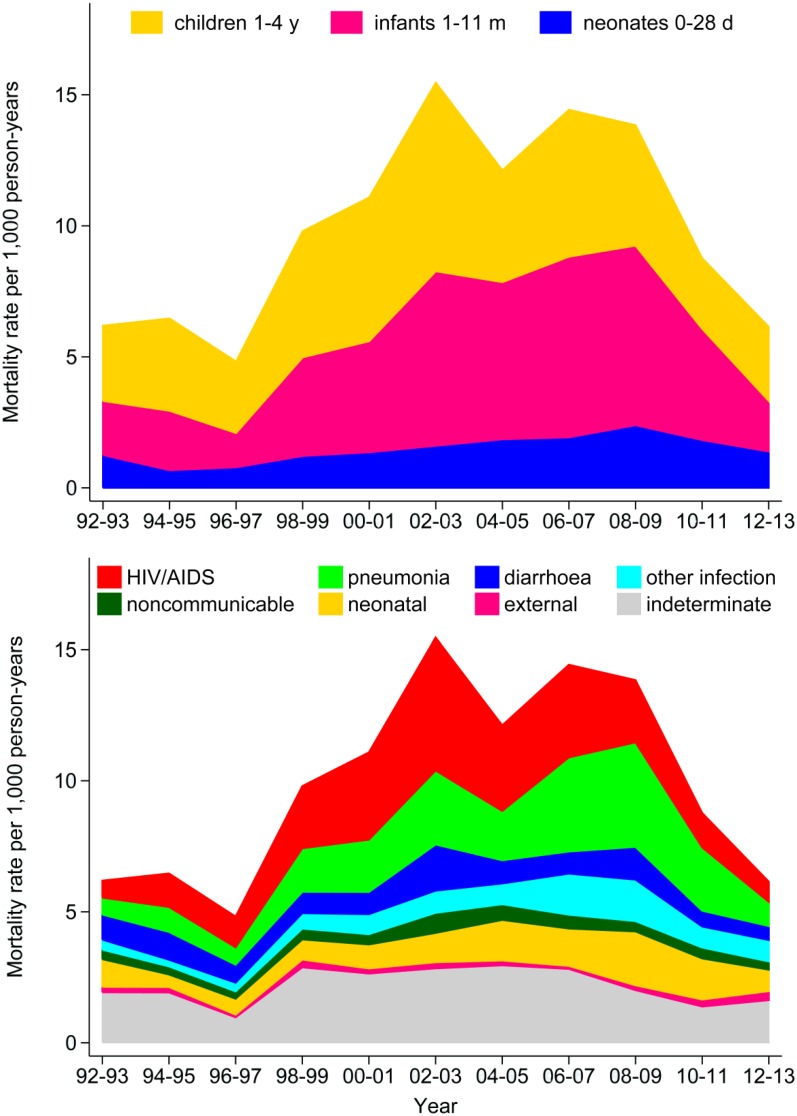
Under-five mortality rates at the Agincourt site from 1992 to 2013, by age group and by cause of death category.

Fig 2 shows the dynamics of under-five mortality (split into neonatal, infant and 1–4-year-old groups) and corresponding causes of death over the period 1992 to 2013. This shows very similar levels at the beginning and end, while observing a major increase and then decrease in the intervening period, during which mortality more than doubled. The biggest component of under-five mortality occurred in the 1–11 month age range, particularly during the height of the mortality peak. Cause-specific analysis showed that HIV/AIDS (20.2%) and associated pneumonia (22.7%) were the leading overall causes of death, particularly during the mortality peak from 1998 to 2009. Marked neonatal mortality persisted throughout, showing a modest increase over time. By 2012–13, under-five mortality had fallen to 6.1 per 1,000 person-years (48 per 1,000 live births) and infant mortality to 3.3 per 1,000 person-years (26 per 1,000 live births).

This is a highly informative insight into the South African situation over two critical decades, despite confirming South Africa’s failure to tick the MDG4 box for achieving a two-thirds reduction in under-five mortality from 1990 to 2015. While the overall mortality findings are congruent with the national estimates in Fig 1, the cause-specific data also clearly demonstrate the massive burden of HIV-related mortality in Agincourt, that South Africa has faced in general, and the country’s considerable success in recent years in counteracting the epidemic.

Most of the excess mortality in Fig 2 was attributable either directly to HIV/AIDS or indirectly related via concomitant infectious causes. Increased infectious disease mortality among HIV-positive children is well documented [13], and in Agincourt accounted for more than doubling of all-cause under-five mortality during the peak years of the epidemic. Unfortunately, at national level, due to a highly conflicted policy environment in the 1990s, there were considerable delays in the public sector rollout of anti-retroviral therapy (ART) and prevention of mother-to-child transmission (PMTCT) as the epidemic escalated [14,15]. This lack of timely PMTCT roll-out delayed the downturn of the mortality epidemic among under-fives. Nevertheless, as seen here and also demonstrated in a national modelling study [16], once PMTCT and ART programmes were firmly in place, rapid progress in reducing child mortality was achieved.

Had the HIV epidemic not been such a major influence on mortality in South Africa during her first two decades of democracy, there would undoubtedly have been extensive assessment of the effects of political change on mortality [17], As it is, with the HIV epidemic becoming manifest at almost the same time as the democratic transition [18], it is very difficult to conclude as to what might have happened to South African mortality trends in the absence of HIV.

## What Are the Implications for Judging Global Target Achievements?

As the world transitions from MDGs to SDGs, much is being made of the numbers of countries achieving MDG targets. Countries unambiguously meeting the relatively arbitrary MDG criteria will be justly proud of their achievements. Going forward, attention will shift to SDG targets. However, our illustrations of the South African situation clearly demonstrate the need to allow room for detailed consideration of the considerable achievements that may have been made in various places, but which do not fully meet specific criteria, both for current MDG and future SDG assessments. Population health and well-being is not primarily a tick-box exercise but depends on the efforts of thousands of health workers to make differences in their own locations and contexts, which aggregate to substantial effects. Provided good data are collected and promptly made available, changes can be understood and interpreted in detail. An essential component of this will involve paying more attention to routinely determining cause of death [19].

For South Africa, the headline news is that child mortality is now back to where it was in the early 1990s. Although at first sight this may not seem like a major achievement, on closer examination it clearly has been so, in terms of reversing the disastrous effects of the HIV epidemic on mortality patterns during a period of emerging democracy following decades of apartheid. This has indeed been a “successful failure” in terms of MDG outcomes. Further progress towards the SDG target will require widespread improvements in socio-economic conditions and health systems functionality, with national leadership commitment.

## Ethics

The Agincourt HDSS was reviewed and approved by the Committee for Research on Human Subjects (Medical) of the University of the Witwatersrand (protocol M960720 and M081145). Community consent from civic and traditional leadership was secured at the start of surveillance in 1992 and is reaffirmed from time to time, and informed verbal consent is obtained at individual and household level at each annual follow-up visit. A record is kept of the household respondent who consented to be interviewed as well as the responsible fieldworker.

**The specific Agincourt dataset relating to these findings is the INDEPTH Data Repository,** http://www.indepth-ishare.org/index.php/catalog/54.

## Supporting Information

S1 Dataset Dataset of 2,929 records relating to 1,997 individual children who died before their fifth birthday from 1992 to 2013 in the Agincourt population. (XLSX)

## Abbreviations

ART: anti-retroviral therapy
MDG: Millennium Development Goal
PMTCT: prevention of mother-to-child transmission
SDG: Sustainable Development Goal
